# The Role of Parvalbumin Interneuron GIRK Signaling in the Regulation of Affect and Cognition in Male and Female Mice

**DOI:** 10.3389/fnbeh.2021.621751

**Published:** 2021-03-26

**Authors:** Eden M. Anderson, Skyler Demis, Hunter D’Acquisto, Annabel Engelhardt, Matthew Hearing

**Affiliations:** Department of Biomedical Sciences, Marquette University, Milwaukee, WI, United States

**Keywords:** G protein-gated inwardly-rectifying K^+^, GIRK, parvalbumin, prefrontal cortex cognition, affect

## Abstract

Pathological impairments in the regulation of affect (i.e., emotion) and flexible decision-making are commonly observed across numerous neuropsychiatric disorders and are thought to reflect dysfunction of cortical and subcortical circuits that arise in part from imbalances in excitation and inhibition within these structures. Disruptions in GABA transmission, in particular, that from parvalbumin-expressing interneurons (PVI), has been highlighted as a likely mechanism by which this imbalance arises, as they regulate excitation and synchronization of principle output neurons. G protein-gated inwardly rectifying potassium ion (GIRK/Kir3) channels are known to modulate excitability and output of pyramidal neurons in areas like the medial prefrontal cortex and hippocampus; however, the role GIRK plays in PVI excitability and behavior is unknown. Male and female mice lacking GIRK1 in PVI (Girk1^flox/flox^:PVcre) and expressing td-tomato in PVI (Girk1^flox/flox^:PV^Cre^:PVtdtom) exhibited increased open arm time in the elevated plus-maze, while males showed an increase in immobile episodes during the forced swim test (FST). Loss of GIRK1 did not alter motivated behavior for an appetitive reward or impair overall performance in an operant-based attention set-shifting model of cognitive flexibility; however it did alter types of errors committed during the visual cue test. Unexpectedly, baseline sex differences were also identified in these tasks, with females exhibiting overall poorer performance compared to males and distinct types of errors, highlighting potential differences in task-related problem-solving. Interestingly, reductions in PVI GIRK signaling did not correspond to changes in membrane excitability but did increase action potential (AP) firing at higher current injections in PVI of males, but not females. This is the first investigation on the role that PVI GIRK-signaling has on membrane excitability, AP firing, and their role on affect and cognition together increasing the understanding of PVI cellular mechanisms and function.

## Introduction

Cognitive flexibility is the ability to adapt behavior in response to changing environmental contingencies and is a critical component of everyday life. As such, impairments in flexibility increase susceptibility to negative life events (e.g., stress), reduce emotional control, and promote the development of maladaptive behaviors that can disrupt the capacity of individuals to engage in their lives effectively (Lange et al., 2017; Waltz, 2017; Gabrys et al., 2018). Neuropsychiatric disorders such as major depressive disorder (MDD), obsessive-compulsive disorder (OCD), autism, and schizophrenia share common symptomology including cognitive inflexibility, reduced inhibitory control, and impaired working memory (Marazziti et al., 2010; Moghaddam and Javitt, 2012; Diamond, 2013; Etkin et al., 2013; Remijnse et al., 2013; Dajani and Uddin, 2015); however what contributes to these impairments is not well understood.

Optimal regulation of affect (i.e., emotion) and flexible decision-making require a balance of excitation and inhibition (E/I) in numerous cortical and subcortical circuits including the medial prefrontal cortex (mPFC; Pantazopoulos et al., 2006; Kehrer et al., 2008; Yizhar et al., 2011; Gandal et al., 2012a; Murray et al., 2015). Pathological alterations in GABA transmission, in particular, that of parvalbumin-expressing interneurons (PVI), has been highlighted as a likely mechanism by which E/I imbalances and associated symptomology arises (Cardin et al., 2009; Sohal et al., 2009; Gandal et al., 2012b; Murray et al., 2015; Wöhr et al., 2015; Kim et al., 2016a; Ferguson and Gao, 2018). Analysis of postmortem human brain tissues revealed decreased expression of parvalbumin and parvalbumin mRNA in patients affected by schizophrenia or autism (Hashimoto et al., 2003; Curley and Lewis, 2012; Lewis, 2014; Hashemi et al., 2017). Similarly, loss of parvalbumin in rodents promotes autism- (Wöhr et al., 2015) and depression-like symptoms (Fogaça and Duman, 2019), together suggesting a role for parvalbumin and PVI in related neuropsychiatric disorder symptomatology.

Within cortical regions such as the mPFC, PVIs are powerful coordinators of network activity (Markram et al., 2004; Klausberger and Somogyi, 2008; Rudy et al., 2011; Kepecs and Fishell, 2014), with fast-spiking PVIs comprising approximately 50% of cortical interneurons (Kawaguchi and Kubota, 1997). PVIs target the soma and perisomatic compartments of principle output pyramidal neurons where they regulate excitation and synchronize firing (Celio and Heizmann, 1981; Kubota and Kawaguchi, 1994; Atallah et al., 2012; Kvitsiani et al., 2013; Hu et al., 2014; Ferguson and Gao, 2018) to orchestrate cortical information flow (Sohal et al., 2009; Murray et al., 2015; Kim et al., 2016a). In the mPFC, PVIs are highly recruited by afferent excitatory signaling; however intrinsic cellular properties (e.g., membrane excitability) dictate how cells respond to this excitatory drive.

G protein-gated inwardly rectifying potassium ion (GIRK/Kir3) channels produce a slow hyperpolarizing current which modulates neuron excitability and spike firing (Hearing et al., 2013; Marron Fernandez de Velasco et al., 2015; Nimitvilai et al., 2017) acting through inhibitory G protein-coupled receptors including GABA_B_R (Glaaser and Slesinger, 2015; Luján and Aguado, 2015). The role of mPFC and forebrain GIRK channels on pyramidal neuron excitability and behavioral outcomes has been established (Hearing et al., 2013; Victoria et al., 2016); however, while GIRKs are known to reside in PVIs and likely contribute to GABA_B_R-mediated signaling in the hippocampus (Booker et al., 2013), their role on mPFC PVI excitability and output is not known. Increasing evidence suggests that drugs that target G protein inhibitory signaling may serve as clinically relevant therapeutic strategies to treat both cognitive and affect-related symptoms (Mombereau et al., 2004; Slattery et al., 2005; Gandal et al., 2012a; Kumar et al., 2013; Lecca et al., 2016). Thus, identifying key modulators of this signaling and how it varies across cell-types remains an important step. Similarly, while PVIs have become a target of recent therapies, there is a need to better understand the cellular mechanisms that regulate their function before progress can be made in this regard (Hu et al., 2014). Accordingly, this study focuses on inhibitory signaling mediated by PVI GIRK channels by determining the role this signaling has on the output of PVI in the prelimbic cortex and its relevance to prefrontal cortex-dependent regulation of affect and cognitive flexibility in both male and female mice.

## Materials and Methods

### Animals

*Girk1^flox/flox^* stock mice were generated as described (Signorini et al., 1997; Kotecki et al., 2015), and donated by Dr. Kevin Wickman (University of Minnesota). Male *Girk1^flox/flox^* mice were bred with female mice purchased from Jackson Laboratories expressing cre recombinase in parvalbumin-expressing neurons (B6.129P2-Pvalbtm^1(cre)Arbr^/J; Stock No:017320) then backcrossed to create *Girk1^flox/flox^*. Female *Girk1^flox/flox^* mice positive for PVcre were then bred with male mice purchased from Jackson Laboratories expressing tdtomato in parvalbumin-positive neurons (C57BL/6-Tg(Pvalb-tdtomato)15Gfng/J; Stock No: 027395) and backcrossed to generate experimental *Girk1^flox/flox^* mice hemizygous for PVcre and PVtdtomato. For all experiments, *Girk1^flox/flox^* without cre recombinase present were used as controls whereas *Girk1^flox/flox^* with cre recombinase was used as the experimental group. For behavioral experiments, a subset of control and experimental mice also expressed tdtomato in parvalbumin-positive neurons. For electrophysiology experiments, mice also expressed tdtomato in parvalbumin-positive neurons. Mice were housed in a temperature and humidity-controlled room with a 12/12 h light/dark cycle with food and water available *ad libitum* except throughout attention set-shifting and the progressive ratio test. Male and female experimental mice were PD78 ± 2 days at the start of visual cue testing. Behavioral procedures were conducted in the light phase. Experiments were approved by the Institutional Animal Care and Use Committee at Marquette University.

### Behavioral Testing Timeline

**Figure 1:**
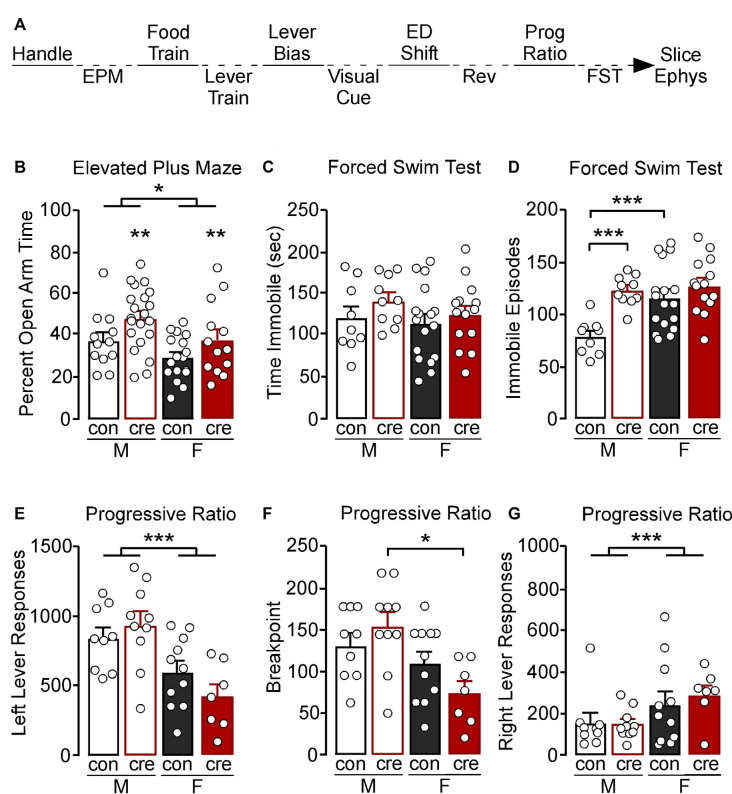
**(A)** Experimental timeline. Mice were handled for at least 3 days then tested in the elevated plus-maze (EPM), food and lever trained, followed by an assessment for a lever bias. Mice were then tested in a visual cue test, an extradimensional shift (ED shift), and a reversal (REV) test, followed by an assessment of motivation in a progressive ratio (Prog Ratio) test. Mice were then tested in the forced swim test (FST). Whole-cell slice electrophysiology was conducted in a subset of mice; however not all mice that had slice electrophysiology underwent behavior. **(B)** Males spent a greater percentage of time in the open arm compared to females (*p < 0.05). Cre-positive mice had a significant increase in percent open arm time (**p < 0.01). **(C)** Male and female control and cre-positive mice had a similar amount of immobile time in the FST. **(D)** Female control mice had a greater number of immobile episodes compared to male control mice (***p < 0.001). Male cre-positive mice had a greater number of immobile episodes compared to control males (***p < 0.001). **(E)** During the progressive ratio, males had a greater number of left lever active presses compared to females (***p < 0.001). **(F)** Female cre-positive mice had a significantly lower breakpoint compared to male cre-positive mice (*p < 0.05). **(G)** Female mice had a greater number of right lever inactive presses compared to males, regardless of condition (***p < 0.001).

Mice were handled for 3 days then tested in the elevated plus-maze (EPM), followed by attention set-shifting training and testing, progressive ratio, and forced swim test (FST; Figure 1A). The battery of behavioral tests was conducted in this order to reduce potential effects of stress on later behavioral tests. EPM was run first, followed by attention set-shifting and progressive ratio during which mice were food-deprived which has been used as a stressor during chronic unpredictable stress protocols and has been shown to elevate corticosterone levels in rats (for reviews see Carr, 2002; Antoniuk et al., 2019). To food-deprive, mice were also single housed which has been shown to elicit endocrine changes (for review see Mumtaz et al., 2018) and early life isolation has been shown to influence EPM behavior in rats (Wright et al., 1991). FST was assessed last because inescapable swim stress has been used as a physical and psychological stressor to elicit a coping response (for review see Molendijk and de Kloet, 2015; de Kloet and Molendijk, 2016). Despite efforts to run behavioral tests from least to most stressful, it should be noted that carryover effects from one behavioral test to the next cannot be excluded. A subset of mice were then used for slice electrophysiology; however, not all mice used for slice electrophysiology underwent behavioral assessments.

### Elevated Plus Maze

Mice were tested for anxiety-like behaviors using the EPM as previously described (Anderson et al., 2019) under low light conditions (50 lux at the center of the maze). AnyMaze (Stoelting Company) tracking software was used to record and analyze behavior. Percent time in the open arms was calculated as total time in the open arms divided by total time in the maze.

### Attention Set-shifting

Attention set-shifting training and tests were conducted in operant conditioning chambers (Med Associates, Inc.) and were modified and based on methods previously described (Brady and Floresco, 2015). Following EPM, mice were food-deprived to 85–90% of their free-feeding weight. For all attention set-shifting training and testing, correct responses were rewarded with the presentation of 50% liquid Ensure^®^ diluted in tap water. During *food training*, a fixed ratio 1 schedule was used, whereby a response on either the left or the right lever resulted in the delivery of a reward. The initial food training session was 3 h in length and repeated daily until the mouse earned at least 50 rewards. The next day, mice had 30 min to receive at least 50 rewards and this training session was also repeated daily until the mice did so.

During *lever training*, retractable levers were pseudorandomly presented with no more than two consecutive extensions of each lever. Mice were required to press the lever within 10 s of lever extension to receive a reward; the absence of a lever press was deemed as an omission. Each lever training session consisted of 90 trials (45 of each the left and right lever), with each trial followed by a 20 s time out. Mice were required to reach criterion of ≤5 omissions total on two consecutive days to move forward. Once the lever training criterion was reached, *lever bias* was assessed during which both the right and left lever were presented and reinforced on a fixed ratio of one for a total of seven trials. If a mouse did not have ≤5 omissions for two consecutive days within 15 days of lever training, training was ceased and the mouse did not progress through testing (*N* = 12).

The following day, *visual cue testing* was conducted until 150 trials or 10 consecutive correct responses were reached with a minimum of at least 30 trials. If the criterion was not reached on the first day of testing, a second or third day of testing was conducted. During visual cue testing, an illuminated cue light was presented above either the left or right lever in a pseudorandomized order. A response on the lever underneath the illuminated cue light resulted in reward delivery and a 20 s time out; incorrect responses resulted in only the time out. Omissions were counted as stated above and were not counted towards a trial to criterion (i.e., neither correct nor error) when data was analyzed but counted towards the daily 150 trials. The next day, *extradimensional shift testing* was conducted, during which the reinforced lever was always the opposite lever of the previously assessed lever bias. The cue light was presented in a manner/order similar to that during the visual cue test; however, it was not associated with the correct response. Mice were run in the extradimensional shift test until 10 consecutive responses or 150 trials were conducted with a minimum of 30 trials. The day after the extradimensional shift test criterion was reached (i.e., 10 consecutive correct responses), reversal testing was conducted during which the reinforced lever was always that of the previously assessed lever bias; however, the cue light was presented identically to that used during the visual cue test. For set-shifting experiments, all tests for a mouse were excluded if >15 omissions in a given test were recorded as this may reflect mechanical issues, motoric, or motivational issues and may influence responding on subsequent tests.

For further behavioral assessment, the types of errors were analyzed based on previously published methods (Brady and Floresco, 2015). Tests were divided into blocks of 16 trials, not including trials with omitted responses. For visual cue testing, *initial errors* were errors that were made within each block until there were less than six in a single block. Once there were less than six errors in a single block, errors in all subsequent blocks were characterized as *regressive errors*. For the extradimensional set shift test, tests were divided into bins of 16 trials. Errors that were made based on the previous visual cue test rule used, such that the response was made on the lever under the illuminated cue light, were considered* perseverative* until less than six errors in a single bin were made. Errors in the next bin and subsequent bins were considered regressive errors. *Never reinforced errors* were those that were incorrect but the response was not on the lever underneath the illuminated cue light. For the reversal test, perseverative and regressive errors were assessed as described above; however, errors were considered perseverative until less than 10 errors in a bin were made. Separately, errors were also divided into errors that were made towards the cue light distractor and away from the cue light distractor. Response latencies were measured as the amount of time from the extension of the lever until a response was made.

### Progressive Ratio

Following attention set-shifting, mice were then tested for motivation using liquid Ensure^®^ as the reinforcer. Responses on the left lever were reinforced whereas responses on the right lever were inactive and resulted in no consequences. Responses required to obtain each subsequent reward progressively increased. The schedule of reinforcement was (5e^0.2*n^)-5 (Richardson and Roberts, 1996) and testing lasted for a total of 90 min. Following progressive ratio testing, food was returned *ad libitum*.

### Forced Swim Test

A transparent glass beaker 7′′ in diameter was filled with 25 ± 2°C to a depth that prevented the mouse from touching the bottom. Mice were individually placed in the water and habituated for 2 min. Behavioral assessment was recorded during the subsequent 4 min during which immobile time and number of episodes were tracked. Following testing, mice were immediately dried and kept in a warm holding cage. Behaviors were recorded using a side-mounted camera and assessed using AnyMaze tracking software. Immobility sensitivity was set at 85% and a minimum of 250 ms to be counted as an immobility episode.

### Slice Electrophysiology

Mice were anesthetized with isoflurane (Henry Schein), decapitated, and the brain removed and put in ice-cold 95% O_2_ 5% CO_2_ oxygenated sucrose solution (229 mM sucrose, 1.9 mM KCl, 1.2 mM NaH_2_PO_4_, 33 mM NaHCO_3_, 10 mM glucose, 0.4 mM ascorbic acid, 6 mM MgCl_2_, and 0.5 mM CaCl_2_). Coronal slices (300 μm) containing the mPFC were collected using a Leica VT1000S vibratome. Slices were immediately incubated at 31°C for 10 min in a solution containing 119 mM NaCl, 2.5 mM KCl, 1 mM NaH_2_PO_4_, 26.2mM NaHCO_3_, 11 mM glucose, 0.4 mM ascorbic acid, 4 mM MgCl_2_, and 1 mM CaCl_2_. Slices were then removed, allowed to cool to room temperature, and incubated further for a minimum of 35 min.

Whole-cell recordings were performed as previously described (Hearing et al., 2013; Anderson et al., 2019). Oxygenated ACSF (125 mM NaCl, 2.5 mM KCl, 25 mM NaHCO_3_, 10 mM glucose, 0.4 mM ascorbic acid, 1.3 mM MgCl_2_, and 2 mM CaCl_2_) was gravity perfused at a temperature of 29–33°C at a flow rate of ~1.5–2.5 ml/min. Sutter Integrated Patch Amplifier (IPA) with Igor Pro (Wave Metrics, Inc.) was used for the data acquisition software. Recordings were filtered at 2 kHz and sampled at 5 kHz. Layer 5/6 PVI were identified based on the presence of td-tomato fluorescence. For all recordings, adequate whole-cell access (*Ra* < 40 MΩ) was maintained. Borosilicate glass pipettes were filled with 140 mM K-Gluconate, 5.0 mM HEPES, 1.1 mM EGTA, 2.0 mM MgCl_2_, 2.0 mM Na_2_-ATP, 0.3 mM Na-GTP, and 5.0 mM phosphocreatine (pH 7.3, 290 mOsm). For ML297 recordings, a baseline with <20% fluctuation current was obtained, followed by bath application of 10 μM ML297 (David Weaver, Vanderbilt) in 0.04% DMSO (Sigma–Aldrich). Evoked currents were reversed using bath application of 0.30 mM barium chloride (Thermo Fisher Scientific). For rheobase and action potential (AP) frequency, a 20 pA current-step injection was used (0–300 pA, 1 s current injections). Capacitance, membrane resistance, and resting membrane potential (RMP) were taken as a simultaneous value after obtaining whole-cell access. AP duration was measured as the time to reach half the amplitude for the first action potential. Afterhyperpolarization (AHP) amplitude was measured from the spike threshold equipotential point to the maximum amplitude of the first action potential hyperpolarization. Sample sizes are denoted as *n* for the number of recordings/cells and *N* for the number of mice.

### Statistical Analysis

Data are presented as mean ± SEM. SigmaPlot 11.0 was used to perform statistical analyses. A 2 (male, female) × 2 (control, cre) analysis of variance was used for all comparisons except assessment of progressive ratio breakpoint which violates parametric assumptions, and therefore a Kruskal–Wallis test was used. The Student–Newman–Keuls method for multiple *post-hoc* comparisons was used when applicable. Statistical outliers (±2 SD) from behavioral tests were excluded from analyses (two data points from EPM; five total mice from all attention set-shift tests; one data point from progressive ratio; two action potentials from action potential and afterhyperpolarization analyses). For electrophysiology data, cell-based and animal-based analyses were conducted during which each recording was a single data point or recordings from each animal were averaged and counted as a single data point, respectively.

## Results

### Effects of GIRK1 Knockout in PVI on EPM, Forced Swim Test, and Motivation

Constitutive knockout of GIRK signaling, including channels expressing the GIRK1 subunit, has been shown to alter anxiety- and depression-like behavior, learning, and memory, as well as motivation for appetitive rewards (Pravetoni and Wickman, 2008; Wydeven et al., 2014; Llamosas et al., 2015; Victoria et al., 2016). However, recent work has shown that cell-type-specific ablation of GIRK signaling has unique effects on behavior (Victoria et al., 2016), making straightforward interpretation of these phenotypes difficult. As GIRK channels are known to be present in PVI, and alteration of PV-dependent GABA neuron activity is known to regulate affect and cognitive control (Sohal et al., 2009; Rossi et al., 2012; Sparta et al., 2014; Murray et al., 2015; Kim et al., 2016b; Page et al., 2019), we aimed to determine whether GIRK1-dependent signaling in PVI is necessary for normal affect, motivation, and cognitive control.

To examine the behavioral relevance of PVI GIRK1 signaling, we assessed affect-related behavior using the EPM and FST. Individual differences in locomotor activity were controlled for by assessing the percent of time spent in the open arm divided by the total time assessed. A main effect of sex was identified, with males having increased percent time in the open arm compared to females (*F*_(1,59)_ = 5.91, *p* = 0.018). There was also a main effect of condition, with cre-positive mice having increased percent open arm time compared to control mice (*F*_(1,59)_ = 7.05, *p* = 0.010); however no sex by condition interaction was observed (*F*_(1,59)_ = 0.07, *p* = 0.789; Figure 1B).

During the FST, there were no differences in the total time spent immobile (sex: *F*_(1,46)_ = 0.87, *p* = 0.356; condition: *F*_(1,46)_ = 1.91, *p* = 0.174; interaction: *F*_(1,46)_ = 0.20, *p* = 0.657; Figure 1C). There was, however, a significant sex by condition interaction when comparing number of immobile episodes during testing (*F*_(1,46)_ = 4.78, *p* = 0.034), with male cre-positive mice having significantly more immobile episodes than control counterparts (*p* < 0.001), whereas there were no differences between conditions in female mice (*p* = 0.200). Female controls also had increased immobile episodes compared to the male controls (*p* < 0.001), whereas male and female cre-positive mice did not differ (*p* = 0.619; Figure 1D).

To examine if the loss of GIRK1 in PVI impacts motivation and to control for any potential differences observed in our set-shifting experiments (see below), we assessed responding for an appetitive liquid reward (Ensure^®^) using a progressive ratio model. Responses on the left reinforced lever, the breaking point at which the mouse would no longer respond, and the right nonreinforced lever were recorded. Males had an overall greater number of left lever presses compared to females, regardless of condition, whereas controls and cre-positive mice had similar number of responses (sex: *F*_(1,33)_ = 18.50, *p* < 0.001; condition: *F*_(1,33)_ = 0.22, *p* = 0.640; interaction: *F*_(1,33)_ = 2.43, *p* = 0.128; Figure 1E). A Kruskal–Wallis nonparametric test detected a significant difference on breakpoint during progressive ratio (*H*_(3)_ = 10.76, *p* = 0.013), with breakpoints in cre-positive males greater than cre-positive female mice, and no other significant *post-hoc* comparisons (Figure 1F). Notably, similar to the left lever, a main effect of sex was observed for responding on the right non-reinforced lever (*F*_(1,33)_ = 5.44, *p* = 0.026). In this case, females displayed a higher number of presses; however no effect of condition or interaction was observed (condition: *F*_(1,33)_ = 0.15, *p* = 0.702; interaction: *F*_(1,33)_ = 0.26, *p* = 0.614; Figure 1G).

### Effects of PVI GIRK1 Knockout on Cognitive Flexibility

**Figure 2:**
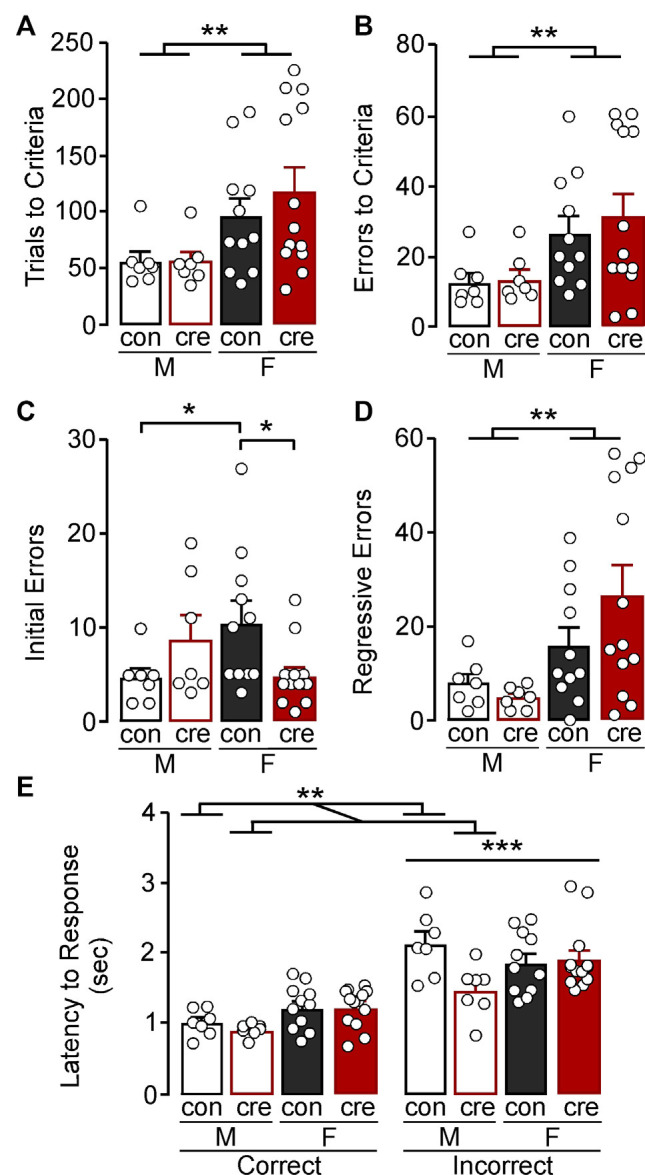
Visual cue test. **(A)** Females took more trials to reach the criterion compared to males (**p < 0.01). **(B)** Females made more errors to reach criterion compared to males (**p < 0.01). **(C)** Female control mice made more initial errors compared to male control mice while female cre-positive made fewer errors compared to female control mice (*p < 0.05). **(D)** Females made more regressive errors compared to males (**p < 0.01). **(E)** The latency to make an incorrect response was higher than the latency to make a correct response (***p < 0.001). Cre-positive male mice, regardless of response type, took less time to respond compared to control mice (**p < 0.01).

Our recent work has shown that disruption of GIRK1 signaling in mPFC prelimbic pyramidal neurons impairs cognitive performance in an attentional set-shifting model of cognitive flexibility (Anderson et al., 2020). Given prior research highlighting a role for PVI activity in cognitive control, we next assessed whether GIRK1 signaling in PVI also influences cognitive control. Before attention set-shift testing, mice had to have two consecutive days with ≤5 omissions during lever training during which there was no effect of sex (*F*_(1,34)_ = 0.94, *p* = 0.340), condition (*F*_(1,34)_ = 1.64, *p* = 0.210), nor an interaction (*F*_(1,34)_ = 1.59, *p* = 0.216; data not shown) on the number of days to reach this criterion. Once mice passed lever training criterion and were assessed for a side bias, testing in the visual cue was conducted. There was an effect of sex on the total trials to reach criterion during the visual cue test (*F*_(1,34)_ = 8.32, *p* = 0.007) but no effect of condition (*F*_(1,34)_ = 0.42, *p* = 0.522) or a sex by condition interaction (*F*_(1,34)_ = 0.41, *p* = 0.527; Figure 2A). Similarly, there was an effect of sex on the total errors made to reach criterion during the visual cue test (*F*_(1,34)_ = 8.32, *p* = 0.007) but no effect of condition (*F*_(1,34)_ = 0.31, *p* = 0.583) nor a sex by condition interaction (*F*_(1,34)_ = 0.41, *p* = 0.710; Figure 2B).

In a more refined investigation into errors, we divided the error type based on errors made before the mice received less than six errors in one 16 trial bin (i.e., initial errors) and those after (regressive errors), the latter of which reflects an inability to maintain the rule strategy. For initial errors, there was significant sex by condition interaction (*F*_(1,34)_ = 7.72, *p* = 0.009; Figure 2C) with *post-hoc* comparisons indicating no significant difference between male control and cre-positive mice (*p* = 0.154), whereas female cre-positive mice had fewer initial errors compared to control mice (*p* = 0.012). Notably, control females had significantly more initial errors compared to control males (*p* = 0.027), whereas male and female cre-positive mice did not differ (*p* = 0.116). Examination of regressive errors showed no main effect of condition (*F*_(1,34)_ = 0.61, *p* = 0.441) or a condition by sex interaction (*F*_(1,34)_ = 1.98, *p* = 0.169); however females had significantly more regressive errors compared to males (*F*_(1,34)_ = 9.09, *p* = 0.005; Figure 2D). Together, further analyses of the error type revealed that regressive errors drive the increased errors to criterion in females, while assessment of the initial errors indicate female control mice have greater difficulty at the beginning of the test compared to male controls and female cre-positive mice.

The speed of processing and general motor function of each mouse can be measured by taking the average latency to respond after the lever was presented (Brady and Floresco, 2015). A 2 (sex) × 2 (condition) × 2 (response type) ANOVA was used to compare latency to respond during the visual cue test. There was a main effect of response type with latency for mice to respond being significantly greater for incorrect compared to correct responses (*F*_(1,68)_ = 76.88, *p* < 0.001; Figure 2E). There was also significant sex by condition interaction (*F*_(1,68)_ = 6.01, *p* = 0.017) with *post-hoc* comparisons indicating that male cre-positive mice took less time to respond compared to male controls (*p* = 0.006), while females, regardless of condition, had similar response latencies (*p* = 0.758). There were no other significant interactions (sex by response type: *F*_(1,68)_ = 1.06, *p* = 0.307; condition by response type: *F*_(1,68)_ = 2.09, *p* = 0.153; sex by condition by response type: *F*_(1,68)_ = 3.06, *p* = 0.085).

**Figure 3:**
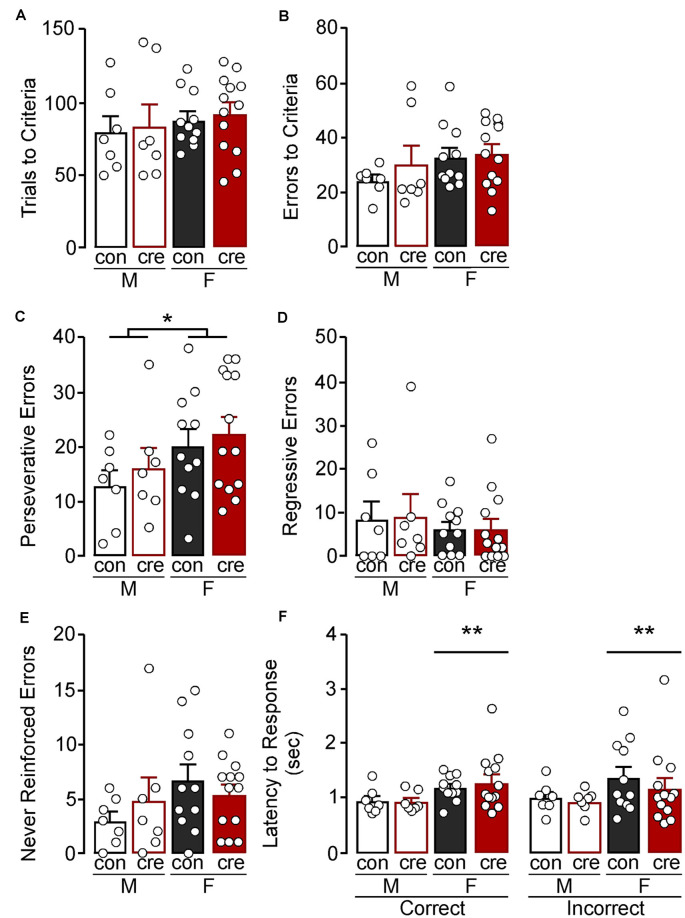
Extradimensional shift test. **(A)** Trials criterion and **(B)** errors to criterion were similar for all groups. **(C)** Females made more perseverative errors compared to males (*p < 0.05) while the number of **(D)** regressive and **(E)** never reinforced errors were similar for all groups. **(F)** Females took longer to respond compared to males (**p < 0.01) regardless of condition and response type.

During the extradimensional shift test, there were no differences in trials to reach criterion comparing sex (*F*_(1,34)_ = 0.84, *p* = 0.365), condition (*F*_(1,34)_ = 0.21, *p* = 0.653), or sex by condition (*F*_(1,34)_ = 0.00, *p* = 0.978; Figure 3A). There were also no differences in errors to reach criterion comparing sex (*F*_(1,34)_ = 2.22, *p* = 0.145), condition (*F*_(1,34)_ = 0.79, *p* = 0.381), or sex by condition (*F*_(1,34)_ = 0.37, *p* = 0.551; Figure 3B). However, females had overall more perseverative errors than males (*F*_(1,34)_ = 4.34, *p* = 0.045), while there were no differences of condition on number of perseverative errors (*F*_(1,34)_ = 0.71, *p* = 0.404) or a sex by condition interaction (*F*_(1,34)_ = 0.03, *p* = 0.882; Figure 3C). There was no main effect of sex (*F*_(1,34)_ = 0.72, *p* = 0.402), main effect of condition (*F*_(1,34)_ = 0.01, *p* = 0.910), nor a sex by condition interaction (*F*_(1,34)_ = 0.01, *p* = 0.094) on regressive errors during the extradimensional shift test (Figure 3D). There were also no differences on number of errors made that had previously never been reinforced (sex: *F*_(1,34)_ = 2.27, *p* = 0.141; condition: *F*_(1,34)_ = 0.03, *p* = 0.857; interaction: *F*_(1,34)_ = 1.28, *p* = 0.265; Figure 3E). There was a significant effect of sex on latency to respond, regardless of condition and response type, with females taking longer to respond compared to males (*F*_(1,68)_ = 7.39, *p* = 0.008; Figure 3F) but no other differences (condition: *F*_(1,68)_ = 0.21, *p* = 0.645; response type: *F*_(1,68)_ = 0.08, *p* = 0.772; sex by condition: *F*_(1,68)_ = 0.00, *p* = 0.974; sex by response type: *F*_(1,68)_ = 0.00, *p* = 0.973; condition by response type: *F*_(1,68)_ = 0.64, *p* = 0.427; sex by condition by response type: *F*_(1,68)_ = 0.26, *p* = 0.614).

**Figure 4:**
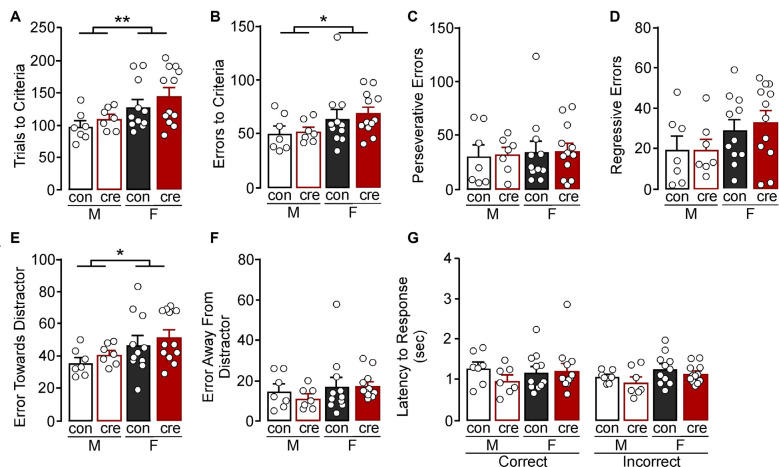
Reversal test. **(A)** Females took more trials (**p < 0.01) and **(B)** errors to criterion (*p < 0.01) compared to males. **(C)** All groups had a similar number of perseverative and **(D)** regressive errors. **(E)** Females made more errors towards the cue distractor compared to males (*p < 0.05). **(F)** The number of errors away from the distractor was similar for all groups. **(G)** All groups had similar latency to respond, regardless of response type.

During the reversal test, females took more trials to reach criterion compared to males (*F*_(1,33)_ = 8.03, *p* = 0.008), but there were no differences comparing conditions (*F*_(1,33)_ = 1.59, *p* = 0.216) or a sex by condition interaction (*F*_(1,33)_ = 0.04, *p* = 0.849; Figure 4A). Similarly, females had more errors during the reversal test compared to males (*F*_(1,33)_ = 4.54, *p* = 0.041); however there was no difference comparing the conditions (*F*_(1,33)_ = 0.24, *p* = 0.630) or a sex by condition interaction (*F*_(1,33)_ = 0.07, *p* = 0.793; Figure 4B). There were no differences in perseverative errors (sex: *F*_(1,33)_ = 0.16, *p* = 0.695; condition: *F*_(1,33)_ = 0.02, *p* = 0.882; sex by condition: *F*_(1,33)_ = 0.00, *p* = 0.969; Figure 4C) or regressive errors (sex: *F*_(1,33)_ = 3.94, *p* = 0.056; condition: *F*_(1,33)_ = 0.12, *p* = 0.727; sex by condition: *F*_(1,33)_ = 0.14, *p* = 0.709; Figure 4D). Errors were next analyzed as being either toward or away from the cue distractor. There was a significant effect of sex on errors towards the distractor (*F*_(1,33)_ = 5.30, *p* = 0.028) with females making more errors towards the distractor (Figure 4E), but there were no differences between the two conditions (*F*_(1,33)_ = 1.11, *p* = 0.299) or a sex by condition interaction (*F*_(1,33)_ = 0.00, *p* = 0.988). There were also no differences in number of errors made away from the distractor (sex: *F*_(1,33)_ = 1.43, *p* = 0.241; condition: *F*_(1,33)_ = 0.24, *p* = 0.629; interaction: *F*_(1,33)_ = 0.31, *p* = 0.582; Figure 4F). Finally, there were no differences in latency to make a response during the reversal test (sex: *F*_(1,66)_ = 2.17, *p* = 0.146; condition: *F*_(1,66)_ = 1.81, *p* = 0.184; response type: *F*_(1,66)_ = 0.44, *p* = 0.510; sex by condition: *F*_(1,66)_ = 1.00, *p* = 0.322; sex by response type: *F*_(1,66)_ = 0.45, *p* = 0.506; condition by response type: *F*_(1,66)_ = 0.00, *p* = 0.944; sex by condition by response type: *F*_(1,66)_ = 0.79, *p* = 0.377; Figure 4G). The extradimensional shift and reversal tests suggest that cre-positive mice have similar cognitive flexibility to control mice, and also reveal that females tend to persist on the initial visual cue test rule (i.e., increased perseverative errors during the extradimensional shift test and increased errors towards distractor during the reversal test) despite the changing contingencies.

### Characterization of GIRK1 Knockout in Prelimbic Cortex PVI

**Figure 5:**
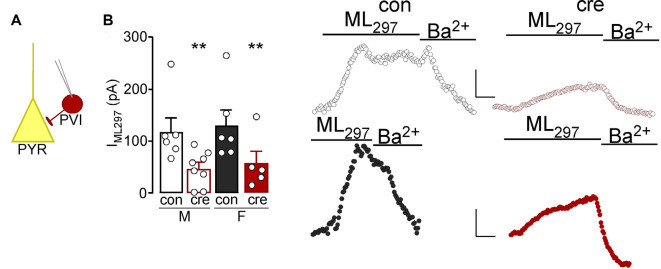
**(A)** Whole-cell slice electrophysiology was used to record from parvalbumin interneurons (PVI) labeled with td-tomato in the prelimbic cortex. **(B)** The current induced by bath application of ML297 was lower in PVI from cre-positive mice (right representatives) compared to those from control mice (left representatives; **p < 0.01). Scale bar: 50 pA/180 s.

Past work has identified an important role for GIRK1 signaling in hippocampal PVI (Booker et al., 2013). Given known contributions of the prelimbic region of the mPFC in regulating the aforementioned behaviors, we confirmed the presence of GIRK1 in prelimbic PVI and characterized knockout in *Girk1^flox/flox^* mice. To facilitate the electrophysiological evaluation of PVI neurons in PVCre:*Girk1^flox/flox^* mice, we crossed this line with transgenic mice expressing tdtomato under the control of the PV promoter. To determine if the knockout was reducing GIRK1 in PVI, voltage-clamped whole-cell slice recordings were performed to assess GIRK1-specific changes in somatodendritic currents using the GIRK1 selective agonist, ML297 (Wydeven et al., 2014; Figure 5A). Bath application of ML297 produced an outward current (I_ML297_) that correlated with a decrease in input resistance (not shown) and was blocked by subsequent application of barium chloride (0.3 mM). Comparison across sex and condition (con vs. cre) showed that ML297-mediated currents were not different in PVI from males vs. females (*F*_(1,21)_ = 0.26, *p* = 0.616) nor was there a sex by condition interaction (*F*_(1,21)_ = 0.00, *p* = 0.972). PVI recorded from cre-positive mice showed a significant reduction in ML297-induced current compared to PVI from control mice (*F*_(1,21)_ = 10.23, *p* = 0.004; Figure 5B) indicative of a reduction in GIRK1 signaling (male control *n* = 6/*N* = 4; male cre *n* = 8/*N* = 4; female control *n* = 6/*N* = 5; female cre *n* = 5/*N* = 3). Similarly, animal-based analysis during which data points were averaged for each animal show no significant effect of sex (*F*_(1,12)_ = 0.89, *p* = 0.364) nor a sex by condition interaction (*F*_(1,12)_ = 0.00, *p* = 0.951); however there was a main effect of condition (*F*_(1,12)_ = 4.83, *p* = 0.048). Similar to initial findings, recordings from cells in cre-positive mice had a significant reduction in ML297-induced current compared to cells from cre-negative mice indicating a reduction in GIRK1 signaling.

**Figure 6:**
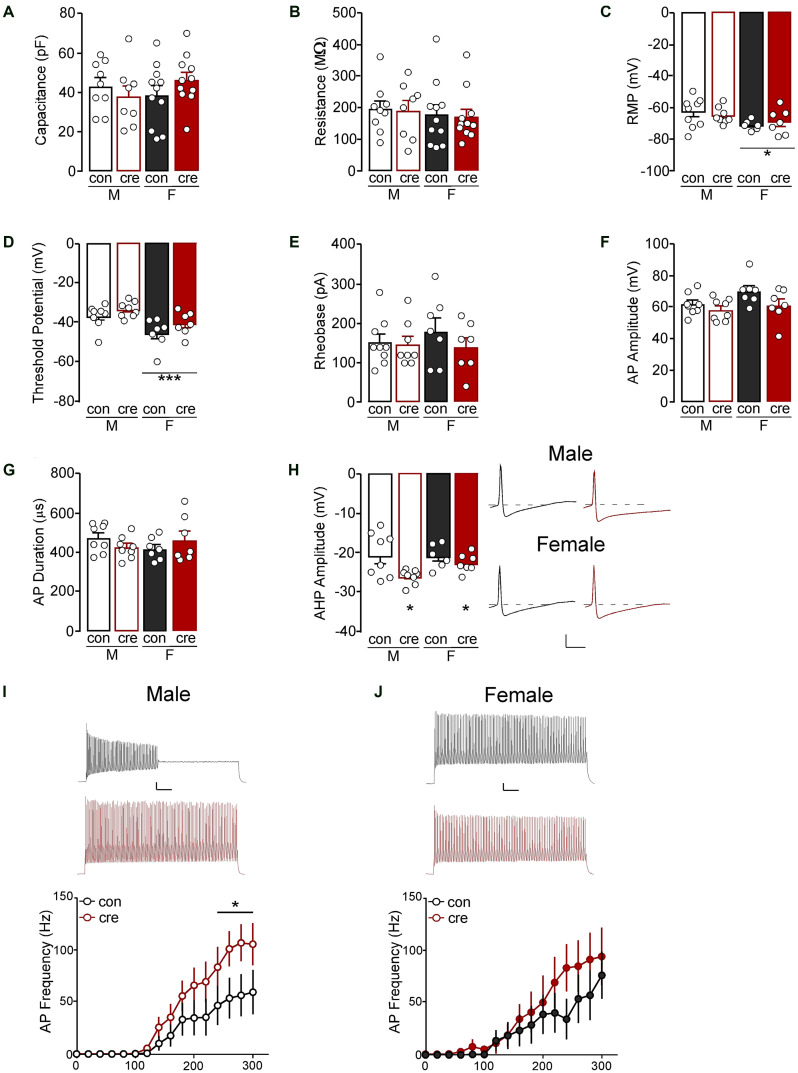
**(A)** The capacitance and **(B)** resistance of PVI neurons were similar for all groups. **(C)** The resting membrane potential (RMP) of PVI from females, regardless of condition, were more negative than those from males (*p < 0.05). **(D)** The threshold potential before firing an action potential was also more negative in PVI from females compared to males (***p = 0.001). **(E)** The current required to evoke an action potential was similar for all groups. **(F)** The action potential (AP) amplitude and **(G)** duration was similar for all groups. **(H)** Afterhyperpolarization (AHP) amplitude for the first action potential from cre-positive PVI, regardless of sex, was more negative compare to PVI from cre-negative mice (*p < 0.05; scale: 20 mV/5 ms). **(I)** The number of action potentials fired at 240–300 pA was higher in PVI from cre-positive male mice compared to control male mice (*p < 0.05; scale 10 mV/100 ms, traces at 300 pA). **(J)** PVI had similar action potential frequency in cre-positive and control female mice (scale 10 mV/100 ms, traces at 300 pA).

To determine how loss of GIRK1 signaling impacts membrane properties we next accessed capacitance, membrane resistance, RMP, threshold to fire an action potential, rheobase, amplitude and duration of initial action potential, AHP amplitude, and spike firing frequency in response to increasing current injections. For cell-based analyses, there were no differences in capacitance (sex: *F*_(1,35)_ = 0.22, *p* = 0.641; condition: *F*_(1,35)_ = 0.05, *p* = 0.826; interaction: *F*_(1,35)_ = 2.10, *p* = 0.157; Figure 6A) or resistance (sex: *F*_(1,35)_ = 0.43, *p* = 0.518; condition: *F*_(1,35)_ = 0.05, *p* = 0.821; interaction: *F*_(1,35)_ = 0.00, *p* = 0.980; Figure 6B; male control *n* = 9/*N* = 6; male cre *n* = 8/*N* = 4; female control *n* = 11/*N* = 5; female cre *n* = 11/*N* = 5). RMP was more negative in females compared to males (*F*_(1,26)_ = 5.13, *p* = 0.032) however there was no main effect of condition (*F*_(1,26)_ = 0.02, *p* = 0.898) nor a sex by condition interaction (*F*_(1,26)_ = 0.61, *p* = 0.442; Figure 6C; male control *n* = 9/*N* = 6; male cre *n* = 8/*N* = 4; female control *n* = 6/*N* = 4; female cre *n* = 7/*N* = 4). The threshold to fire an action potential was also more negative in females compared to males (*F*_(1,26)_ = 13.46, *p* = 0.001) with no main effect of condition (*F*_(1,26)_ = 3.86, *p* = 0.060) nor a sex by condition interaction (*F*_(1,26)_ = 0.09, *p* = 0.773; Figure 6D; male control *n* = 8/*N* = 6; male cre *n* = 8/*N* = 4; female control *n* = 7/*N* = 4; female cre *n* = 7/*N* = 4). Rheobase (i.e., the amount of current injected for the cell to fire an action potential) did not differ based on sex or condition (sex: *F*_(1,27)_ = 0.21, *p* = 0.651; condition: *F*_(1,27)_ = 1.02, *p* = 0.322; interaction: *F*_(1,27)_ = 0.59, *p* = 0.449; Figure 6E; male control *n* = 9/*N* = 6; male cre *n* = 8/*N* = 4; female control *n* = 7/*N* = 4; female cre *n* = 7/*N* = 4). The action potential amplitude (sex: *F*_(1,26)_ = 3.51, *p* = 0.072; condition: *F*_(1,26)_ = 4.16, *p* = 0.052; interaction: *F*_(1,26)_ = 0.88, *p* = 0.356; Figure 6F) and duration at 50% the amplitude did not differ between groups (sex: *F*_(1,26)_ = 0.07, *p* = 0.797; condition: *F*_(1,26)_ = 0.00, *p* = 0.988; interaction: *F*_(1,26)_ = 2.75, *p* = 0.109; Figure 6G; male control *n* = 8/*N* = 6; male cre *n* = 8/*N* = 4; female control *n* = 7/*N* = 4; female cre *n* = 7/*N* = 4). There was a significant effect of condition on the afterhyperpolarization amplitude, with cre-positive mice having greater afterhyperpolarizations compared to control mice (*F*_(1,25)_ = 7.23, *p* = 0.203). However, there were no differences in sex (*F*_(1,25)_ = 1.36, *p* = 0.255) nor a sex by condition interaction (*F*_(1,25)_ = 1.71, *p* = 0.203; Figure 6H; male control *n* = 8/*N* = 6; male cre *n* = 8/*N* = 4; female control *n* = 6/*N* = 3; female cre *n* = 7/*N* = 4). Lastly, during the current-step injection in PVI from males, there was a significant current by condition interaction (*F*_(15, 225)_ = 2.01, *p* = 0.016) with cre-positive cells firing significantly more action potentials at higher currents (240–300 pA) compared to controls (*p* < 0.05; Figure 6I). Conversely, there was no main effect of condition (*F*_(1,12)_ = 0.69, *p* = 0.423) or a condition by current interaction (*F*_(15, 180)_ = 0.75, *p* = 0.735; Figure 6J) in female PVI (male control *n* = 9/*N* = 6; male cre *n* = 8/*N* = 4; female control *n* = 7/*N* = 4; female cre *n* = 7/*N* = 4). Together, these data indicate that loss of GIRK1 does not impact activation threshold but does increase neuronal firing of male PVI.

Animal-based statistics, during which recordings from each animal were averaged, show similar findings to the cell-based statistics. There were no differences in capacitance (sex: *F*_(1,16)_ = 0.03, *p* = 0.867; condition: *F*_(1,16)_ = 0.19, *p* = 0.669; interaction: *F*_(1,16)_ = 2.72, *p* = 0.119) or resistance (sex: *F*_(1,16)_ = 0.13, *p* = 0.728; condition: *F*_(1,16)_ = 0.04, *p* = 0.836; interaction: *F*_(1,16)_ = 0.01, *p* = 0.937). Similar to the cell-based statistics, RMP was more negative in females compared to males (*F*_(1,14)_ = 4.59, *p* = 0.050) however there was no main effect of condition (*F*_(1,14)_ = 0.09, *p* = 0.775) nor a sex by condition interaction (*F*_(1,14)_ = 0.52, *p* = 0.484). The threshold to fire an action potential was also more negative in females compared to males (*F*_(1,14)_ = 13.19, *p* = 0.003) with no main effect of condition (*F*_(1,14)_ = 3.82, *p* = 0.071) nor a sex by condition interaction (*F*_(1,14)_ = 0.73, *p* = 0.406). Rheobase did not differ based on sex or condition (sex: *F*_(1,14)_ = 1.06, *p* = 0.321; condition: *F*_(1,14)_ = 1.71, *p* = 0.212; interaction: *F*_(1,14)_ = 1.06, *p* = 0.321). Unlike cell-based statistics which showed no difference in action potential amplitude, animal-based statistics revealed a significant difference with females having significantly greater amplitude compared to males (sex: *F*_(1,14)_ = 5.94, *p* = 0.029; condition: *F*_(1,14)_ = 2.79, *p* = 0.117; interaction: *F*_(1,14)_ = 1.18, *p* = 0.296). The duration at 50% the amplitude did not differ between groups (sex: *F*_(1,14)_ = 0.07, *p* = 0.794; condition: *F*_(1,14)_ = 0.15, *p* = 0.706; interaction: *F*_(1,14)_ = 2.63, *p* = 0.127). Unlike cell-based statistics, there was no significant effect on the afterhyperpolarization amplitude (sex: *F*_(1,13)_ = 1.30, *p* = 0.275; condition: *F*_(1,13)_ = 4.50, *p* = 0.054; interaction: *F*_(1,13)_ = 1.25, *p* = 0.284). Lastly, during the current-step injection in PVI, there were no significant effects of condition or condition by current interactions in males (condition: *F*_(1,8)_ = 1.80, *p* = 0.217; interaction: *F*_(14, 112)_ = 1.24, *p* = 0.255) or females (condition: *F*_(1,6)_ = 1.83, *p* = 0.225; interaction: *F*_(14, 84)_ = 1.39, *p* = 0.174). Similar to cell-based statistics, these data indicate that loss of GIRK1 does not impact activation threshold however also does not alter neuronal firing in males or females, despite significantly reducing GIRK1 signaling.

## Discussion

The current study evaluated the impact of selectively ablating GIRK channels expressing the GIRK1 subunit in PVI on affect and cognitive flexibility. We found that loss of PVI GIRK1 signaling in males and females increased the percent time spent in the open arm during the EPM, suggestive of reduced anxiety-related behavior. Notably, effects on anxiety-like behavior in EPM or open field were not previously observed with conditional knockout of GIRK2 channels in GABA neurons using GAD-Cre transgenic mice (Victoria et al., 2016), suggesting that more selective targeting of GABA neuron subpopulations may yield discrete changes. Loss of GIRK1 selectively in males also increased immobile episodes during the FST, suggesting an increase in active coping. Although not directly comparable, these findings are in alignment with recent work showing that increased activation of mPFC PVI results in anxiety-like behaviors (Page et al., 2019). Moreover, the lack of effect on motivation during the progressive ratio is in agreement with previous research showing that optogenetic stimulation of mPFC PVI does not alter appetitive reward consumption (Sparta et al., 2014). Past *in vivo* work has shown that increased spike firing of PVI cells reduces the output of pyramidal neurons in the mPFC (Sparta et al., 2014), thus it is possible that any observed changes in behavior, albeit discrete, reflect reductions in prelimbic cortex pyramidal neuron activity.

Given the span of literature highlighting a critical role for PVI in the regulation of mPFC-dependent cortical processing (Ferguson and Gao, 2018) and previously identified roles for GIRK signaling in regulating affect and cognition (Pravetoni and Wickman, 2008; Lazary et al., 2011; Cooper et al., 2012; Yamada et al., 2012; Wydeven et al., 2014; Lecca et al., 2016; Victoria et al., 2016), we predicted that effects of GIRK1 ablation would be evident in measures of cognitive flexibility. These predictions were based on studies using approaches that reduced PVI-dependent signaling, however, the impact of increased PVI output on cortical processing and cognition is far less clear. For example, cre-inducible channelrhodopsin activation of mPFC PVI at high frequencies has been shown to promote delayed alternation impairments (Rossi et al., 2012) and accelerate the extinction of cue-reward behavior (Sparta et al., 2014). Conversely, chemogenetic and optogenetic activation of mPFC PVI does not alter performance in a novel object recognition test of working memory or fear conditioning (Yizhar et al., 2011; Page et al., 2019). In the present study, loss of PVI GIRK1 signaling did not result in behavioral deficits during an overall performance in tests of visual cue-based discriminative learning or flexibility during the extradimensional shift and reversal test—tasks which are dependent on the mPFC (Ragozzino et al., 1999; Floresco et al., 2008) and orbitofrontal cortex (Ghods-Sharifi et al., 2008), respectively. However, during the visual cue test, cre-positive males had a reduced latency to respond compared to their control counterparts, which may be indicative of the increased speed of processing. These findings align with a reduction in attentional processing following PVI silencing (Kim et al., 2016b). Although the alterations in speed of processing did not correspond to differences in the trials or errors to criterion, others have shown that PVI inactivation produces deficits in cognitive flexibility in the water maze (Murray et al., 2015). The outcomes of these studies are difficult to compare to the current study, as they determined the role of acute and intermittent increases in PVI activity, rather than chronically altering PVI activity specifically through reductions in GIRK signaling. Regardless, the present study in combination with past work suggests that while the loss of PVI signaling promotes critical deficits in cognitive control, the effects of increased PVI output are far more complex. In agreement, a recent report (Caballero et al., 2020) has suggested that there may be a threshold of PVI expression through which cortical dysfunction becomes evident. Thus, possibly the loss of GIRK signaling alone does not meet that threshold, as other inhibitory signaling may be decreased to compensate for changes in PVI excitability and output.

### GIRK1 Knockout Effects on PVI Excitability

Past studies have shown that perisomatic inhibition of hippocampal PVI is likely driven by activation of GIRK channels (Booker et al., 2013) and that while GIRK1–3 subunits were present in PVI, GIRK1 was the most prominently expressed. Similarly, our past work has shown that GIRK1-expressing GIRK channels are the primary mediators of GIRK signaling in prelimbic cortex pyramidal neurons, however, to our knowledge this is the first study to assess the role of GIRK signaling in prelimbic cortex PVI. Whole-cell recordings with the selective GIRK1 agonist ML297 showed that GIRK1-containing channels are indeed present in prelimbic PVI and that GIRK1-dependent current do not differ in males or females. The presence of cre-recombinase reduced GIRK1-mediated currents by ~60%, with residual current likely driven by vehicle (DMSO). While GIRK1 knockout produced a leftward shift in the current-spike relationship (increased firing frequency) in PVI from males (albeit only when analyzed using cell-based statistics), to a lesser non-significant extent in females, it unexpectedly did not alter rheobase.

Examination of intrinsic membrane properties using cell-based statistics showed no baseline sex differences or effects of GIRK1 knockout on capacitance, membrane resistance, action potential amplitude, or action potential duration. These data align with previous work from our lab and others showing no significant changes in these measures in prelimbic pyramidal neurons with GIRK1 knockout (Hearing et al., 2013) and a lack of GIRK contribution to the regulation of firing properties (Llamosas et al., 2017; but see Imbrosci and Mittmann, 2013). Conversely, unlike our past findings in prelimbic pyramidal neurons, PVI from cre-positive mice show increased afterhyperpolarization amplitude compared to cre-negative PVI. Further, while not impacted by GIRK1 knockout, RMP was more hyperpolarized in females, and while rheobase did not differ in males and females, more sensitive assessments showed that action potentials are initiated at more hyperpolarized potentials in females. Together, these data highlight effects of GIRK1 ablation on intrinsic physiology that may be both unique to PVI, and also sex-specific, and demonstrate previously uncharacterized sex differences in PVI excitability.

Although analysis of data through the use of animal-based statistics resulted in similar findings for capacitance, membrane resistance, RMP, the threshold to fire an action potential, and action potential duration, there were some inconsistencies. There were no differences in action potential amplitude when analyzed using cell-based statistics, however, animal-based statistics found a significant effect of sex. It should be noted that animal-based statistics assume that each recording is from a similar cell-type. Given noted subpopulations of cells within a given cell-type throughout the brain, including PVI (chandelier vs. basket), and known differences in the electrophysiological characteristics of each cell-type, taking the average of cells from each mouse may increase the risk of a Type 1 error by reducing variability in the statistical analysis. Frequently, each animal contributes one to three recordings within a dataset, therefore, the use of animal-based statistics to average recordings also significantly reduced the sample size which may result in a Type 2 error. Although cell-based statistics revealed a significantly greater afterhyperpolarization amplitude in cre-positive compared to controls, there were no differences when analyzed using animal-based statistics. Similarly, while cell-based statistics revealed greater action potential firing from cre-positive mice compared to controls in males, there were also no differences when analyzed using animal-based statistics. The lack of findings using animal-based statistics may be the result of decreased power due to reductions in sample size from averaging all recordings within each animal.

The lack of a consistent effect on traditional measures of membrane excitability (rheobase, current-spike relationships), as well as lack of a robust behavioral phenotype, may reflect a variety of factors. First, the knockout of PVI GIRK-signaling was already in effect during critical development periods which may have led to compensatory changes in non-GIRK effectors more readily modulating membrane excitability. Although a viral vector to specifically target PVI GIRK-signaling is not readily available, it would be beneficial for future research to target PVI GIRK channels specifically during adulthood to reduce the potential for compensation of other signaling during development. Further, neuronal GIRK channels can be homo- and heterotetrameric complexes formed primarily by the assembly of GIRK1, GIRK2, and GIRK3 subunits (Karschin et al., 1996; Hearing et al., 2013), it is possible that loss of GIRK1 leads to upregulation of homotetrameric GIRK2-expressing channels. However, such a phenomenon has not been observed with constitutive and conditional knockout of GIRK1 or GIRK2 in other cell-types in areas such as the hippocampus, prefrontal cortex, and ventral tegmental area (Hearing et al., 2013; Kotecki et al., 2015; Victoria et al., 2016). Third, mPFC PVIs include basket and chandelier type cells that differ in structure, physiology, and GPCR agonist response (Kawaguchi and Kubota, 1997; Booker et al., 2013). Thus, decreasing GIRK-signaling in both cell-types may have diminished any opportunity for behavioral changes that may have arisen should GIRK-signaling have been reduced in only one cell-type. Finally, GIRK channels may play a role in regulating membrane excitability in PVI, and differences in membrane properties of GIRK1 channel knockout PVI may become evident by blocking fast synaptic transmission. Similarly, although beyond the scope of these studies, PVI GIRK channels may exhibit differences in coupling efficiency or regulators of G protein signaling (Labouebe et al., 2007). Given these and several other limitations of this study, including a lack of anatomical and temporally precise knockout of GIRK1, future research investigating the role that PVI GIRK1 KO has on postsynaptic Gi-GPCR signaling as well as using more targeted manipulations (i.e., viral-mediated) is warranted.

Relatedly, the global knockout approach used also assumedly resulted in reductions in PVI GIRK1-signaling in brain regions other than the prelimbic cortex which may have resulted in changes to PVI excitability in these regions and alterations in behavior paradigms that were not assessed in the current series of experiments. Unfortunately, data on the role of PVI GIRK-signaling in other brain regions on behavior is nonexistent; however the role of PVI in regions such as the amygdala (e.g., Wolff et al., 2014; Luo et al., 2020), hippocampus (Tucker et al., 2019), and striatum (Monteiro et al., 2018) are well-studied.

### Sex Differences in Cognitive Flexibility and Motivation

Although not the main focus of the study, we unexpectedly identified differences in male and female performance across the set-shifting task that were largely independent of GIRK ablation. During the initial visual cue test, females required more trials and errors to reach the criterion compared to males, regardless of genotype. In controls, the increased number of errors in females reflects an equal balance between initial and regressive errors. However, loss of PVI GIRK appears to shift this towards an increase in regressive error types and a reduction in initial errors, suggesting an impaired ability to maintain a newly learned rule.

During the extradimensional shift, while females do not require more trials to reach criterion than males, they perseverate more on the rule associated with the prior day visual cue test (they continue to follow the cue light) and take longer to respond compared to males. Females also require more trials to reach criterion in a test of reversal learning. Further investigation of the error types shows a greater number of errors on the lever underneath the cue (i.e., more errors towards the distractor) indicating that they are still perseverating on the visual cue rule, despite learning a new rule during the extradimensional shift. To our knowledge, this is the first study to investigate sex differences in an operant based attentional set-shifting model of cognitive flexibility. It should be noted that although gonadal hormones influence cognition (Luine and Frankfurt, 2020; Taxier et al., 2020), the estrous cycle does not appear to influence attention set-shifting performance (Workman et al., 2013), and these sex differences may not be replicated in C57BL/6J mice. The day following the reversal test, motivation for an Ensure reward was measured using a progressive ratio test during which only the left lever was reinforced. Although male mice had a significantly greater number of left lever active responses, females exhibited greater responses on the right inactive lever. As progressive ratio testing only occurred on a single day, the increased right lever responses in females may suggest a lack of task understanding with a shift to right lever responding at higher breakpoints. Although interesting future directions arise from these measurable sex differences in a variety of behaviors, it should be noted that the efficiency of the cre-recombinase knockdown was not measured in the current study. Recombination in the PVI:cre strain has been noted in >90% of cells (Jackson Laboratory) however there are examples of incomplete recombination (i.e., cre mosaicism) and differences in efficiency in males and females in other cre strains (Jackson Laboratory) indicating that the potential of sex differences in cre efficiency related to the current findings should not be disregarded.

Findings from the current study suggest that PVI GIRK1 signaling does not mediate membrane excitability to the extent that it does in principle pyramidal neurons and may differentially impact firing frequency in males and females. Further, while reductions in PVI GIRK1 signaling influenced anxiety-like behaviors, effects on cognitive performance were more nuanced. While outside the scope of this study, the demonstration that GIRK channels are present in prelimbic PVI requires further investigation into their function, as past work has shown that differences in subunit expression and coupling dictate responsivity to various Gi-coupled GPCR agonists, and thus may inform future drug therapies targeting GIRKs or Gi-coupled GPCRs. Further, the current findings have provided unexpected insight into how biological sex impacts cognitive processing associated with an operant-based model of cognitive flexibility which may have important implications for treating pathological deficits in cognitive control.

## Data Availability Statement

The raw data supporting the conclusions of this article will be made available by the authors, without undue reservation.

## Ethics Statement

The animal study was reviewed and approved by Marquette University Instituational Animal Care and Use Committee.

## Author Contributions

EA and MH designed, discussed, planned all experiments, and wrote the manuscript. EA, AE, SD, HD’A, and MH performed experiments and analyzed data. All authors contributed to the article and approved the submitted version.

## Conflict of Interest

The authors declare that the research was conducted in the absence of any commercial or financial relationships that could be construed as a potential conflict of interest.
